# Microstructure (EBSD-KAM)-Informed Selection of Single-Powder Soft Magnetics for Molded Inductors

**DOI:** 10.3390/ma18215016

**Published:** 2025-11-04

**Authors:** Chang-Ting Yang, Yu-Fang Huang, Chun-Wei Tien, Kun-Yang Wu, Hung-Shang Huang, Hsing-I Hsiang

**Affiliations:** 1 Material and Chemical Research Laboratories, Industrial Technology Research Institute, Hsinchu 310401, Taiwan; chrisyang@itri.org.tw (C.-T.Y.); kanahuang@itri.org.tw (Y.-F.H.); 2 Department of Resources Engineering, National Cheng Kung University, Tainan 70101, Taiwan; rookietian41@gmail.com; 3 New Materials Research & Development Department, China Steel Corporation, Kaohsiung 81233, Taiwan; 172759@mail.csc.com.tw (K.-Y.W.); 160184@mail.csc.com.tw (H.-S.H.)

**Keywords:** soft magnetic composites (SMCs), FeSiCr, reduced iron powder (RIP), core loss, lattice strain, EBSD, kernel average misorientation (KAM), power inductors

## Abstract

**Highlights:**

**What are the main findings?**

- EBSD KAM links deformation substructure to hysteresis loss (K_h_).
- Particle size and shell control eddy loss (K_e_); fine SiO_2_-coated Fe lowers K_e_.
- FeSiCr shows the highest μ_i_ despite the lowest density; coarse size reduces pinning.
- DC-bias L retention: CIP > RIP > CIP-P > FeSiCr at 15 A (67.9 > 55.7 > 48.8 > 33.2%).

**What is the implication of the main finding?**

- Microstructure-informed map guides single-powder selection for inductors.
- Balance μi, loss, bias, and corrosion via size and shell chemistry.
- RIP offers lower loss, greater durability, and greater sustainability vs. FeSiCr/CIP-P.
- EBSD/KAM becomes a fast-screening metric for bias and loss.

**Abstract:**

This study systematically benchmarks the performance of four single soft magnetic powders—water-atomized Fe–Si–Cr (FeSiCr), silica-coated reduced iron powder (RIP), silica-coated carbonyl iron powder (CIP), and phosphate-coated CIP (CIP-P)—to establish quantitative relationships between powder attributes, deformation substructure, and high-frequency loss for molded power inductors (100 kHz–1 MHz). We prepared toroidal compacts at 200 MPa and characterized them by initial permeability (μ_i_), core-loss (P_cv_(f)), partitioning (P_cv_(f) = K_h_f + K_e_f^2^, K_h_, $K_{e}$: hysteresis and eddy-current loss coefficients), and EBSD (electron backscatter diffraction)-derived microstrain metrics (Kernel Average Misorientation, KAM; low-/high-angle grain-boundary fractions). Corrosion robustness was assessed using a 5 wt% NaCl, 35 °C, 24 h salt-spray protocol. Our findings reveal that FeSiCr achieves the highest μ_i_ across the frequency band, despite its lowest compaction density. This is attributed to its coarse particle size (D_50_ ≈ 18 µm) and the resulting lower intragranular pinning. The loss spectra are dominated by hysteresis over this frequency range, with FeSiCr exhibiting the largest K_h_, while the fine, silica-insulated Fe powders (RIP/CIP) most effectively suppress K_e_. EBSD analysis shows that the high coercivity and hysteresis loss in CIP (and, to a lesser extent, RIP) are correlated with dense, deformation-induced subgrain networks, as evidenced by higher mean KAM and a lower low-angle grain boundary fraction. In contrast, FeSiCr exhibits the lowest KAM, with strain confined primarily to particle contact regions. Corrosion testing ranked durability as FeSiCr ≳ CIP ≈ RIP ≫ CIP-P, which is consistent with the Cr-rich passivation of FeSiCr and the superior barrier properties of the SiO_2_ shells compared to low-dose phosphate. At 15 A, inductance retention ranks CIP (67.9%) > RIP (55.7%) > CIP-P (48.8%) > FeSiCr (33.2%), tracking a rise in effective anisotropy and—for FeSiCr—lower M_s_ that precipitate earlier roll-off. Collectively, these results provide a microstructure-informed selection map for single-powder formulations. We demonstrate that particle size and shell chemistry are the primary factors governing eddy currents (K_e_), while the KAM-indexed substructure dictates hysteresis loss (K_h_) and DC-bias superposition characteristics. This framework enables rational trade-offs between magnetic permeability, core loss, and environmental durability.

## 1. Introduction

The rapid adoption of wide-bandgap (WBG) power semiconductors such as SiC and GaN has pushed switching frequencies and power densities upward, shrinking passive components while imposing tighter limits on magnetic losses and DC-bias stability in power inductors [1,2]. In these regimes, material and device co-design is critical to meet thermal and electromagnetic constraints without sacrificing footprint or reliability [1,2].

Soft magnetic composites (SMCs)—electrically insulated ferromagnetic powders compacted into near-net-shape cores—have emerged as a versatile platform for high-frequency magnetics thanks to their isotropy, high electrical resistivity, and formability [3,4,5]. Among Fe-based SMCs, Fe–Si–Cr (FeSiCr) powders are widely used for molded inductors because they balance saturation flux density, resistivity, and cost, whereas ferrites offer lower loss but lower saturation and more limited DC-bias tolerance [2,3,4,5].

However, FeSiCr cores often exhibit higher core loss than ferrites at comparable flux densities. Mixing FeSiCr with finer, softer powders (e.g., carbonyl iron powder, CIP) can raise packing density and relax domain wall pinning, lowering hysteresis and improving DC-bias performance—an approach increasingly used in molded inductor formulations [2,6,7].

From a sustainability perspective, substituting part of the virgin metallic feed with recycled reduced iron powder (RIP) obtained from steel mill scale aligns with circular-economy goals while potentially lowering the cost and embedded energy relative to fully carbonyl-processed feedstocks [8,9]. RIP can be produced by the direct reduction of mill scale and then surface-modified (e.g., silica/phosphate) for SMC processing; by contrast, CIP is made via the carbonyl route into very fine, spherical powders [10,11].

Despite widespread powder blending in industry, the underlying micro-mechanical origins of loss reduction are not fully quantified. Magnetic core loss is commonly separated into rate-dependent components (hysteresis ∝ f, eddy currents ∝ f^2^, with excess/dynamic contributions), but connecting these macroscopic parameters to microstructural metrics in pressed powders remains challenging [12,13]. Electron backscatter diffraction (EBSD) offers a route to quantify local lattice curvature/strain via Kernel Average Misorientation (KAM), which correlates with geometrically necessary dislocation (GND) density and, by extension, domain wall pinning and hysteresis [11,12,13]. Recent EBSD studies establish the methodological basis for using KAM/GND as proxies for stored strain and defect density in deformed metals, supporting a structure–property link in SMCs [14,15,16,17].

This work (i) systematically compares recycled, silica-coated RIP with commercial CIPs and FeSiCr powder for SMCs and molded inductors, and (ii) employs EBSD to quantify lattice strain via KAM in compacts to establish a direct microstrain–core loss connection.

## 2. Materials and Methods

### 2.1. Materials

Base powder: Water-atomized Fe–Si–Cr (FeSiCr) alloy powder (Advanced Technology & Materials Co., Ltd., Beijing, China), and three insulated soft-Fe powders were evaluated: (i) silica-coated reduced iron powder, RIP, sourced from China Steel Corporation (Kaohsiung, Taiwan); (ii) silica-coated carbonyl iron powder, CIP, from BASF Corporation (Ludwigshafen, Germany); and (iii) phosphate-coated commercial CIP-P (Sintez-CIP, Moscow, Russia) were used as the raw materials. All powders were stored in desiccators before use.

### 2.2. Sample Preparation

#### 2.2.1. Mixing and Granulation

A 2 wt% phenolic resin (with respect to total powder mass) dissolved in acetone was then added dropwise under slow tumbling to achieve incipient wetness and homogeneous granulation. The granulated powders were dried in a ventilated oven and gently passed through a 40-mesh sieve.

#### 2.2.2. Compaction and Curing

Green toroids with an outer diameter (OD) of 12.85 mm and inner diameter (ID) of 7.75 mm were uniaxially pressed at 200 MPa in a lubricated steel die. All compacts were cured at 150 °C for 2 h to cross-link the phenolic binder.

### 2.3. Characterization Methods

#### 2.3.1. Compositional and Physical Analyses

Elemental compositions of Fe, Si, Cr, and P (when present) were measured on pressed powder pellets using X-ray fluorescence spectroscopy (XRF; Rigaku ZSX100S, Tokyo, Japan). Particle-size distributions (PSD) of the as-received powders were characterized by a particle size analyzer (Horiba, LA 350, Kyoto, Japan); ultrasonication was employed to minimize soft agglomeration. Results are reported as D_10_/D_50_/D_90_. Morphologies of the loose powders and of fracture surfaces of selected compacts were characterized by scanning electron microscopy (SEM, HITACHI, SU-5000, Tokyo, Japan).

#### 2.3.2. Structural Analysis (XRD)

Powder X-ray diffraction was performed with a Bruker D2 Phaser (Billerica, MA, USA) using Cu Kα radiation. Scans covered 20–100° 2θ with a 0.02° step and 1–2° min^−1^ rate. Phase identification and qualitative assessment of crystallinity were carried out against ICDD reference patterns.

#### 2.3.3. Measurements of Magnetic Properties

Each toroid was wound with a 25-turn test coil and measured using a Precision Impedance Analyzer (Wayne Kerr, WK6500B, London, UK) at 100 kHz under small-signal excitation (no DC bias). The initial permeability, μ_i_, was obtained from the measured inductance L and the toroid geometry [18]:

μ_i_ = (Lℓ_e_)/μ_0_N^2^A_e_ (1)

ℓ_e_ ≈ π(D_out_ + D_in_)/2 (2)

A_e_ ≈ h(D_out_ − D_in_)/2 (3)

where μ_0_ is the permeability of free space, D_out_ and D_in_ are the measured OD and ID, and h is the thickness.

Total core loss and coercivity (H_c_) were measured with a B–H Analyzer (IWATSU SY-8218, Tokyo, Japan) using sinusoidal excitation from 200 kHz to 1 MHz at 50 mT. The total core loss per unit volume P_cv_(f) comprises hysteresis P_h_, classical eddy current P_e_, and a residual (excess) term P_r_, the last of which is essential only at very low induction levels and at very high frequencies and is, thus, ignorable in this investigation. This two-term model is considered sufficient for these SMC materials, as the insulated particle structure suppresses complex interparticle currents, and the high internal strain (as-pressed) promotes rotational magnetization processes over the domain wall motion associated with anomalous losses. We therefore fit the data to Equation (4) [19],

P_cv_(f) = P_h_ + P_e_ = K_h_f + K_e_f^2^ (4)

where K_h_ and K_e_ are the hysteresis and eddy-current loss coefficients, respectively, and f is frequency.

This linear fitting method is a robust and standard engineering practice for separating the static and dynamic loss coefficients in soft magnetic materials, as demonstrated in numerous studies that apply and modify the Steinmetz and Bertotti frameworks [19,20]. This allowed us to partition the losses for our comparative analysis confidently.

We acknowledge that this two-term model omits the ‘excess’ or ‘anomalous’ loss term. This simplification is justified by the high, as-pressed dislocation densities quantified by our EBSD/KAM analysis, which are expected to promote rotational magnetization processes, thereby minimizing dynamic domain wall motion effects associated with excess loss. Other fundamental mechanisms, such as local demagnetization (from the composite structure) and Barkhausen jumps (from defect pinning), are the physical origins of the static hysteresis and are thus implicitly quantified within the experimentally derived permeability (μ_i_) and hysteresis coefficient (K_h_).

#### 2.3.4. Corrosion Resistance (Salt-Spray)

Salt-spray testing followed a 5 wt% NaCl exposure protocol at 35 °C for 24 h. Samples were cleaned with isopropanol, air-dried, and mounted at an angle of ~15–20° from vertical to prevent pooling. Post-exposure, surfaces were photographed under controlled lighting.

#### 2.3.5. Lattice-Strain Analysis (EBSD-KAM)

Polished cross-sections were prepared from toroids (and selected molded parts) by mounting in epoxy, sequential grinding to 4000-grit SiC, and polishing with 3 µm → 1 µm diamond followed by 0.05 µm colloidal silica (30–45 min, gentle load). Final surfaces were rinsed in ethanol and dried with nitrogen.

EBSD was performed with a HITACHI SU7000 FE-SEM equipped with an Oxford Instruments Symmetry 2 detector at 20 kV accelerating voltage, ~10–12 mm working distance, 70° specimen tilt, and a 0.2 µm step size. Patterns were indexed with FCC/BCC libraries as appropriate; noisy points were cleaned using standard wild-spike removal and confidence-index (CI) standardization. Kernel Average Misorientation (KAM) maps were computed using first-neighbor kernels with a 5° upper misorientation cap to emphasize intragranular lattice curvature. For each specimen, ≥3 regions of interest (≥300 × 300 µm^2^ each) were analyzed to obtain mean KAM and distribution statistics; data with CI < 0.1 were excluded from statistics.

## 3. Results and Discussion

### 3.1. Characterization of Raw Powders

Table 1 shows the elemental compositions of the as-received powders. The FeSiCr powder contains ~88.5 wt% Fe, 5.1 wt% Si, and 6.1 wt% Cr, consistent with a corrosion-tolerant Fe–Si–Cr solid solution. All additive iron powders (RIP and both CIPs) are Fe-rich (Fe > 98 wt%). The detection of Si in the silica-coated grades (RIP and CIP) and P in the phosphate-coated CIP (CIP-P) verifies the intended insulating shells. No deleterious impurities (e.g., S, Cl) were detected above the reporting limit.

Figure S1 shows the volume-based particle-size distribution (PSD) curves, and Table 2 compiles the D_10_/D_50_/D_90_ metrics. FeSiCr is the coarse fraction (D_50_ ≈ 17.8 µm), whereas RIP, CIP, and CIP-P constitute fine fractions (D_50_ ≈ 4–6 µm). The narrower PSD typically observed for carbonyl iron (CIP) reflects its carbonyl synthesis route, while RIP retains a slightly broader spread consistent with reduction-from-oxide processing.

**Figure 1:**
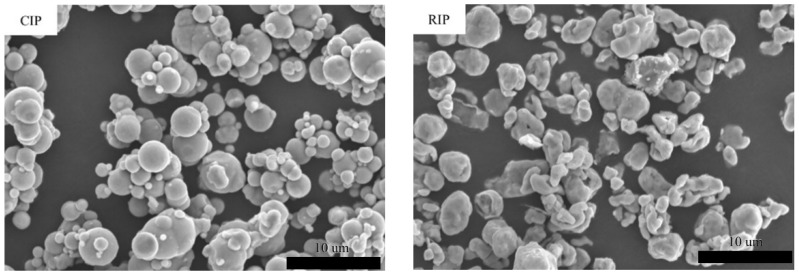

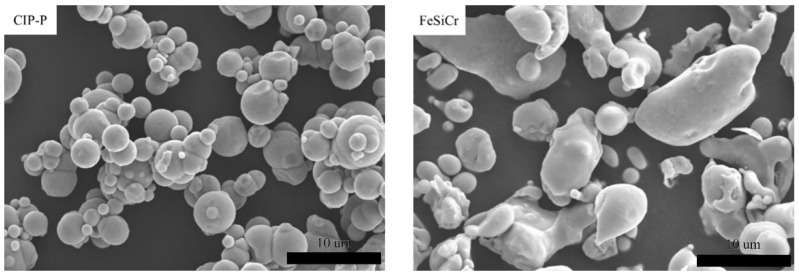
Representative SEM micrographs of the four as-received raw powders.

Figure 1 shows representative SEM images of the raw materials. FeSiCr particles are irregular/angular with faceted asperities (water-atomized morphology). RIP is sub-rounded to irregular with a matte surface and occasional fine satelliting. CIP is near-spherical and smooth, characteristic of the carbonyl route, improving flowability and packing reproducibility. The thin insulating shells are not resolved as continuous films by conventional SEM but are corroborated by XRF (Table 1).

As shown in Figure S2, all powders exhibit three prominent diffraction maxima at approximately 2θ ≈ 45°, 65°, and 83°, which are indexed to α-Fe (bcc) and are characteristic of crystalline soft magnetic iron powders. The FeSiCr powder retains a bcc α-Fe solid-solution signature, and no discernible secondary phases are detected within the XRD detection limit, indicating that the Si/Cr additions remain homogeneously dissolved in the Fe matrix.

Figure S2 compares the diffraction profiles of the four feedstocks before and after uniaxial compaction to assess structure/defect evolution. In the as-received state, all patterns are consistent with α-Fe (bcc) and FeSiCr solid-solution reflections, while the silica/phosphate surface treatments contribute at most a weak amorphous background without distinct crystalline coating peaks. After compaction, the principal Bragg peaks exhibit a measurable broadening (increase in FWHM, β) and, in some cases, slight 2θ shifts characteristic of elastic microstrain. The dislocation density δ scales with $\beta^{2}$ (see Equation (5), where D is crystallite size, θ the Bragg angle, k a constant, and λ the X-ray wavelength) [21]. Thus, the observed post-press broadening indicates an increase in defect density and strain introduced by particle deformation and interparticle locking during pressing. These compaction-induced defects/microstrain provide a mechanistic link to the magnetic trends discussed later (e.g., changes in coercivity and core loss), as higher δ and strain can enhance domain wall pinning while the absence of new crystalline phases confirms that the effects arise from defect/strain fields rather than phase transformations.

(5) $$\delta =\frac{1}{D^{2}}=\frac{\beta^{2}\cos^{2}\theta }{k^{2}\lambda^{2}}$$

To isolate compaction-induced broadening, the effective FWHM was computed as follows:

(6) $$∆\beta =\sqrt{\beta_{\text{compaction}}^{2}-\beta_{\text{powder}}^{2}},$$

with $\beta_{\mathrm{powder}}$ taken from the as-received patterns and $\beta_{\mathrm{compaction}}$ from the compacted specimens (instrumental conditions held constant).

The compaction-induced effective FWHM is FeSiCr (0.26) > RIP (0.20) > CIP (0.19) > CIP-P (0.16). At first glance, this ordering seems at odds with expectations of plasticity—because CIP and RIP (nearly pure Fe) are the most ductile, whereas FeSiCr is stronger/less plastic, one might anticipate smaller peak broadening for FeSiCr. The outcome instead indicates that FWHM is not solely dictated by bulk plasticity but also by how each powder accommodates press-induced strain (e.g., alloy-driven microstrain storage in FeSiCr versus interfacial accommodation in coated/oxide-skinned powders) [22,23]. We therefore defer a full interpretation to a later section, where these XRD results are analyzed alongside EBSD metrics (KAM, LAGB%) and the resulting magnetic consequences (coercivity, hysteresis loss); we also note that solute-induced microstrain in FeSiCr and interfacial oxides can contribute to the observed broadening.

### 3.2. Effect of Iron-Powder Type on Composite Soft Magnetic Properties

Building on the distinct physicochemical and crystallographic attributes of the four iron-based powders (FeSiCr, CIP, RIP, and CIP-P), we now evaluate their consequences for magnetic response, mechanical compactability, and compaction density in pressed toroids (T-cores). The goal is to identify processing–structure–property linkages that guide material selection and process optimization for power magnetics.

**Figure 2:**
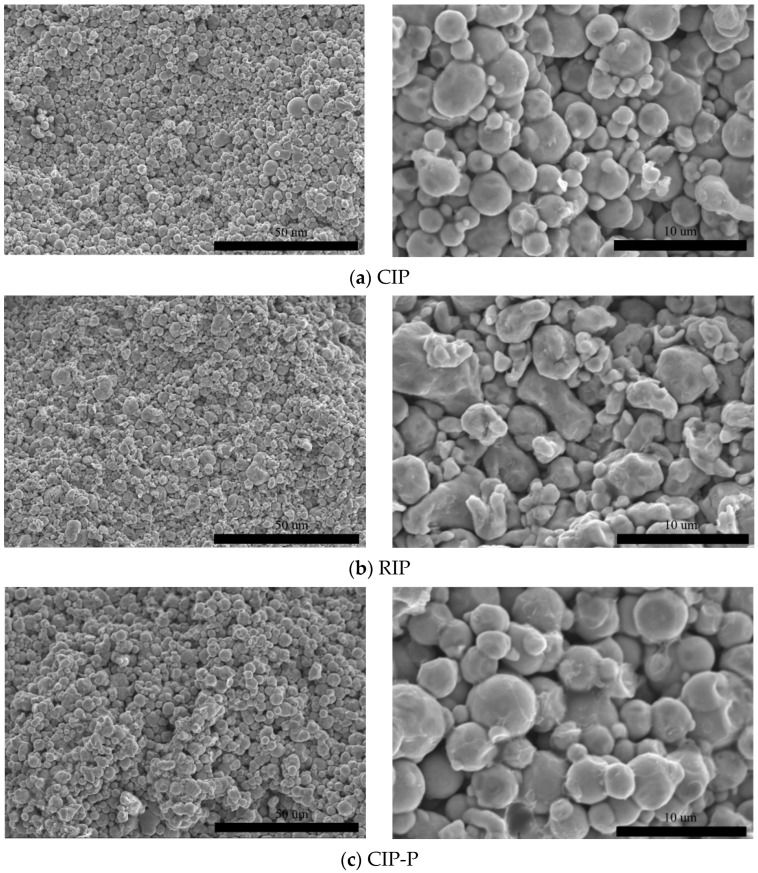

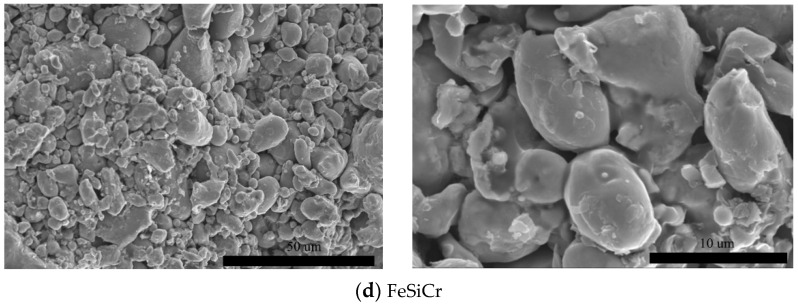
SEM fracture-surface micrographs of T-cores pressed at 200 MPa.

Figure 2 shows the SEM fracture surfaces of compacted T-cores from FeSiCr, CIP, RIP, and CIP-P. Relative to their loose-powder images, particle outlines remain largely recognizable; only limited rounding and modest interparticle plastic flow are evident at this forming pressure. Thus, densification at 200 Mpa is governed primarily by mechanical particle rearrangement rather than extensive plastic flattening, and the original size/morphology contrasts between FeSiCr (coarse, irregular), RIP (sub-rounded/irregular), and CIP/CIP-P (near-spherical) are retained in the green bodies.

The relative green densities for T-cores are summarized in Table 3. CIP and CIP-P achieve the highest densities (74.4% and 73.9%, respectively), RIP is slightly lower (72.3%), and FeSiCr is the lowest (71.7%). This hierarchy reflects powder ductility and flow: the high-purity Fe powders (CIP, RIP, CIP-P; Fe > 98.5%) deform more readily than FeSiCr, whose higher Si/Cr solid-solution content impedes dislocation slip and limits plastic accommodation, thereby reducing densification [22,24]. The small density gap between RIP and CIP is consistent with morphology: RIP’s more irregular shape and slightly poorer flowability yield a lower packing factor than the near-spherical CIP at the same pressure.

**Figure 3:**
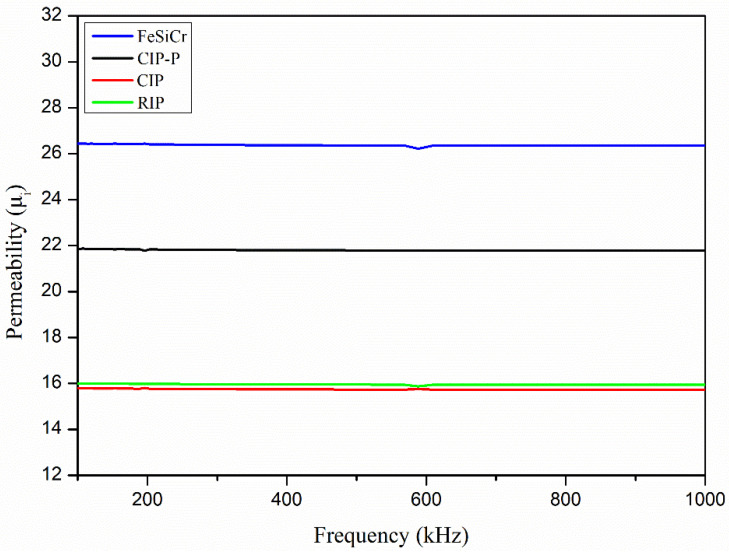
Variation in initial permeability (μ_i_) with the measuring frequency (100–1000 kHz).

Figure 3 compares the initial permeability, μ_i_(f), from 100 kHz to 1 MHz for the toroids. Although FeSiCr has the lowest compaction density (Table 3), it sustains the highest μ_i_ across most of the band. This counterintuitive ordering arises from two microstructural factors. First, FeSiCr’s larger median particle size (D_50_ ≈ 18 µm) reduces the total particle/grain-boundary area and the density of pinning sites, thereby facilitating domain wall motion relative to the finer Fe powders (D_50_ ≈ 4–6 µm). Second, the coated fine-Fe powders—CIP, RIP, and CIP-P—undergo greater plastic straining during pressing and are separated by thin, insulating nonmagnetic shells; the resulting higher dislocation content and nonmagnetic shell weaken interparticle exchange and increase wall pinning, both of which depress μ_i_. Consistent with prior reports, increased interface density and defect content in finer, coated powders lower μ_i_ even when the compaction density is higher [25]. Hence, μ_i_ does not vary monotonically with density; it reflects a balance between packing (which tends to raise μ_i_) and microstructural impediments to domain wall motion (which lower μ_i_), with interparticle insulation and magnetoelastic strain acting as coequal control parameters.

**Figure 4:**
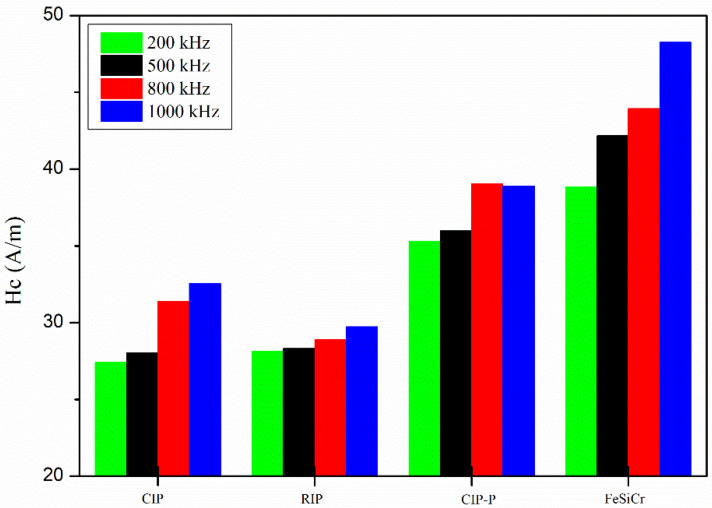
Coercivity (H_c_) versus frequency for FeSiCr, CIP, RIP, and CIP-P.

Figure 4 shows that H_c_ increases with frequency for all powders, consistent with rising dynamic losses and eddy-current fields that elevate the field required for reversal. Coating chemistry plays a clear role: the SiO_2_-insulated powders (CIP and RIP) exhibit lower H_c_ than the phosphate-insulated CIP-P, indicating a distinct interfacial pinning/friction landscape imparted by the insulating shell. In general, larger grains tend to reduce H_c_, but FeSiCr does not show a pronounced decrease in coercivity here. Likely contributors include the following: (i) higher Si/Cr in solid solution acting as point-defect obstacles to domain wall motion; (ii) Cr-rich passivation (e.g., Cr_2_O_3_) at boundaries that further pins walls; and (iii) the lower compaction density (i.e., higher porosity), which introduces volumetric defects that hinder wall motion. Together, these factors offset the grain-size advantage and keep H_c_ comparatively elevated for FeSiCr across the measured band.

**Figure 5:**
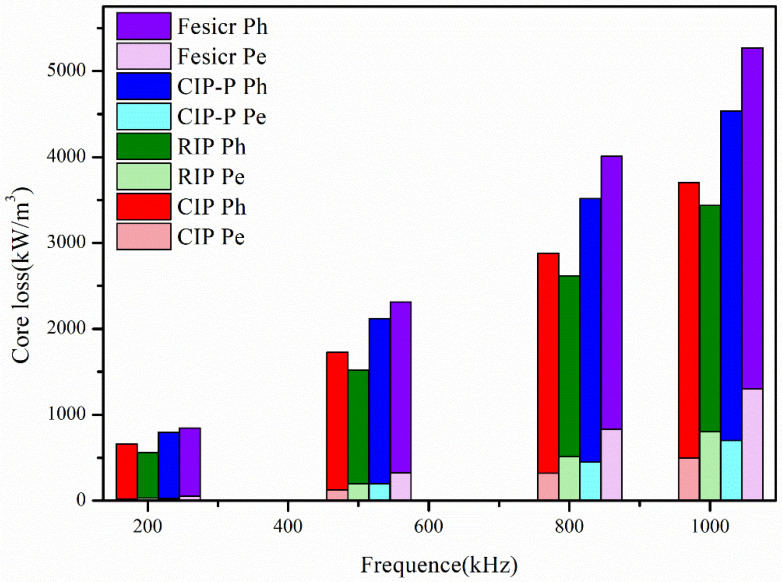
Comparative core loss (hysteresis loss (P_h_), eddy-current loss (P_e_), and total core loss (P_e_ + P_h_) performance of the four powders, measured from 200 kHz to 1000 kHz.

Figure 5 compares the total core loss P_tot_(f) from 200 kHz to 1 MHz at a fixed small-signal induction Bm. Across the band, both CIP and RIP exhibit lower P_tot_ than FeSiCr, indicating better suitability for high-frequency operation. Over 200 kHz–1 MHz, the spectra are hysteresis-dominated: FeSiCr yields the largest K_h_, consistent with its higher H_c_ and stronger domain wall pinning, arising from elevated solute content (Si/Cr), interfacial oxides, and porosity. Among the coated Fe powders, CIP tends to show slightly higher hysteresis than RIP, plausibly reflecting a thicker/more continuous SiO_2_ shell and associated interfacial stresses that increase rotational pinning—an inference supported by the higher Si signal in XRF. The eddy-current term tracks electrical segmentation and characteristic size: FeSiCr (no applied shell, larger D_50_) exhibits the highest eddy-current loss, whereas CIP, RIP, and CIP-P show reduced b due to insulating shells and finer dimensions [26,27]; within this group, CIP typically attains the lowest eddy-current loss, consistent with having the most effective SiO_2_ insulation.

Indeed, while the FeSiCr compact had the lowest density (71.7%), a factor which should theoretically reduce K_e_, its measured K_e_ was still the highest in the set; this confirms that the large particle size is the dominant mechanism governing the eddy-current loss, overwhelming any secondary effects from density variations.

**Figure 6:**
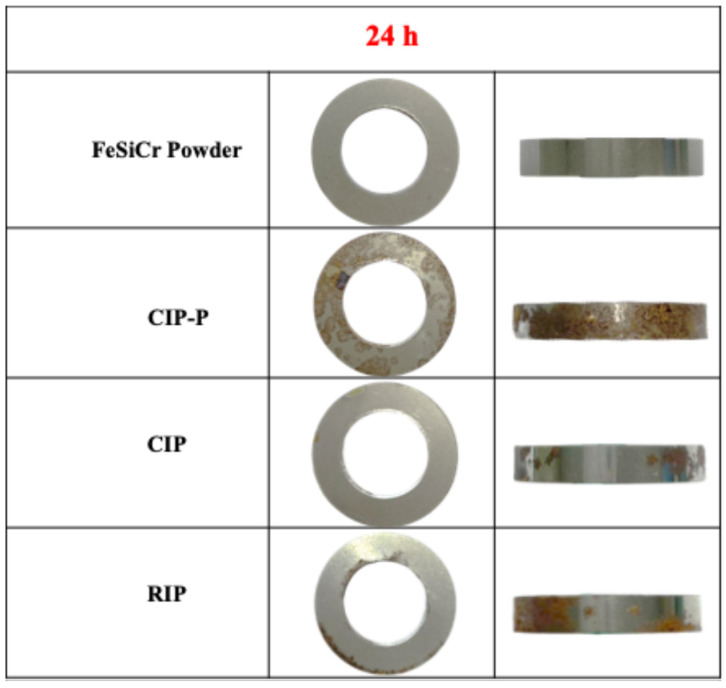
Salt-spray (5 wt% NaCl, 35 °C, 24 h) corrosion snapshots.

Figure 6 summarizes the 24 h salt-spray exposure (5 wt% NaCl at 35 °C) for the T-cores, with visual inspections. The CIP-P cores show the most serious red rust, indicating a limited barrier effectiveness of the phosphate film under continuous chloride challenge. CIP and RIP specimens showed virtually no rust on the top and bottom (loaded) faces, with only trace rust specks on the die-contact sidewall. This behavior is consistent with their silica protective layers, which provide superior barrier performance: on the loaded faces, the surface is denser and the coating remains intact, effectively blocking moisture and chloride ingress. By contrast, the sidewall experiences die-wall friction and shear abrasion during pressing and ejection, which can locally thin or disrupt the silica layer and expose the Fe substrate; concomitant microcracks and open pores act as initiation sites for corrosion [28]. The FeSiCr cores exhibit only slight superficial staining after 24 h; this behavior is consistent with the formation of a dense, adherent Cr_2_O_3_ passivation layer during powder production, which affords robust corrosion resistance even under extended exposure conditions [29,30]. The relatively poorer performance of CIP-P aligns with prior reports that low-dose phosphate treatments (<1 wt%) provide limited, non-durable protection in aggressive salt environments [31]. Overall, the anti-corrosion ranking observed here is FeSiCr ≳ CIP ≈ RIP » CIP-P, supporting the conclusion that SiO_2_ shells (CIP, RIP) provide more durable barrier protection than phosphate (CIP-P), while FeSiCr benefits intrinsically from Cr-rich passivation.

To quantitatively connect microstructure to magnetic performance, we use EBSD-derived metrics as inputs into the loss model *P*(*f*) = *K*_h_*f* + *K*_e_*f*^2^. Specifically, the mean KAM serves as an areal measure of stored plastic strain/GND density; a higher KAM implies denser curvature fields and more domain wall pinning, which raises coercivity (*H*_c_) and thus the hysteresis coefficient (*K*_h_) (at fixed induction, *K*_h_ ∝ *H*_c_). The LAGB fraction complements KAM by quantifying the density and continuity of dislocation walls that act as barriers: at comparable KAM, a larger LAGB share increases the spatial connectivity of pinning sites and tends to further elevate *K*_h_.

**Figure 7:**
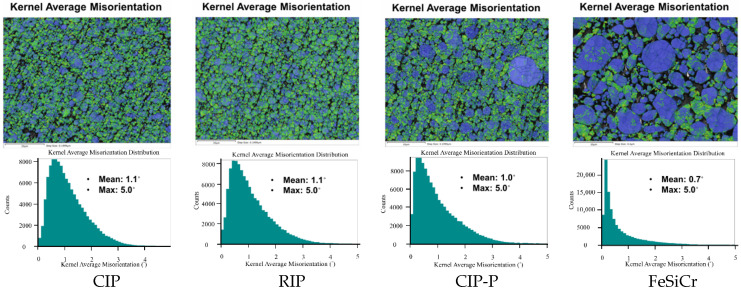
EBSD-derived lattice strain analysis of the four pressed compacts. (**Top row**) Spatial Kernel Average Misorientation (KAM) maps, where warmer colors indicate higher lattice strain. (**Bottom row**) Corresponding KAM-distribution histograms for each material.

Figure 7 shows the Kernel Average Misorientation (KAM) maps (top row) with the corresponding KAM histograms (bottom row) for CIP, RIP, CIP-P, and FeSiCr. All datasets were acquired under identical EBSD conditions (same step size and a 5° KAM cap), and the color scale is fixed across panels, allowing the maps to be directly comparable. Cooler tones (blue) indicate low local lattice curvature (low stored strain), whereas warmer tones (green) denote higher curvature arising from geometrically necessary dislocations (GNDs). For the pure-Fe powders (CIP, RIP, CIP-P), the KAM maps present a fine, nearly continuous mosaic, with extensive green pixels distributed throughout the microstructure. This texture reflects plasticity-dominated compaction: during pressing, ductile Fe particles plastically flow at contact necks and along slip systems, building dense dislocation structures that register as elevated KAM [32]. The histograms reinforce this view—each shows a broad, right-skewed distribution with a long high-misorientation tail. The mean KAM values cluster near 1.0–1.1° (CIP ≈ 1.1°, RIP ≈ 1.1°, CIP-P ≈ 1.0°), indicating that, although RIP has slightly finer grains than CIP2, the level of stored strain is comparable; the governing factor is the extent of plastic accommodation rather than the modest grain-size difference.

By contrast, the FeSiCr compact exhibits large, blue-dominated grains with a higher KAM confined to narrow bands near some boundaries. The histogram is sharply peaked at very low misorientation with a short tail, and the mean KAM is the lowest of the set (≈0.7°). This pattern is consistent with fracture-limited densification: FeSiCr particles tend to crack or microchip under load rather than undergo extensive plastic flow, thereby limiting dislocation generation and lattice curvature [33]. The EBSD evidence, therefore, captures a fundamental processing contrast—ductile Fe powders store strain; FeSiCr does not.

Taken together, the EBSD maps and misorientation histograms provide a microstructural basis for the magnetic trends. In the pure-Fe compacts, higher mean KAM and larger LAGB fractions indicate dense dislocation substructures that pin domain walls, elevating coercivity and the hysteresis contribution to loss. By contrast, the FeSiCr compact exhibits a low-KAM/low-LAGB microstructure—implying fewer strain-generated pinning sites from pressing—yet its hysteresis loss remains high because the elevated Si/Cr solute content introduces chemical pinning (solute drag, local magnetoelastic fields/short-range order) that impedes domain wall motion independent of stored strain. Accordingly, KAM captures the plastic strain component of hysteresis but not the solute chemistry component. Finally, CIP-P shows a slightly lower mean KAM (~1.0°) than CIP/RIP (~1.1°), but the difference is modest; shell chemistry and interface quality can still raise coercivity at similar KAM. Overall, the KAM is informative but not sufficient on its own and must be interpreted alongside the LAGB fraction, solute content, and interparticle insulation when rationalizing loss partitioning.

**Figure 8:**
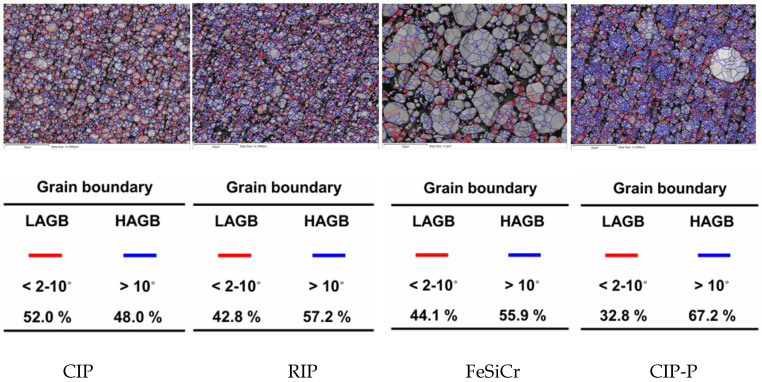
EBSD-identified grain boundary maps for the four pressed compacts. Red lines denote low-angle grain boundaries (LAGB; 2–10°); blue lines mark high-angle grain boundaries (HAGB; >10°).

Figure 8 shows EBSD-identified grain boundaries on orientation maps for the four powder compacts—CIP, RIP, CIP-P, and FeSiCr—and reports the corresponding boundary fractions. Red lines denote low-angle grain boundaries (LAGB; 2–10° misorientation in this dataset), while blue lines mark high-angle grain boundaries (HAGB; >10°). Because all maps were acquired and processed under identical conditions, both the boundary networks and the tabulated LAGB/HAGB percentages are directly comparable.

The CIP compact shows a dense, continuous red network threading through a fine equiaxed microstructure—classic evidence of a deformation-induced subgrain structure. The majority LAGB fraction (≈52%) indicates the extensive storage of geometrically necessary dislocations during pressing, consistent with the higher mean KAM (~1.1°) observed for CIP. Such sub-boundaries provide abundant domain wall-pinning sites, which rationalize the relatively elevated H_c_ and hysteresis loss in this ductile Fe compact [34].

Despite its finer grain size than CIP, RIP exhibits a slightly lower LAGB fraction and a correspondingly higher HAGB fraction. EBSD still reveals a pervasive subgrain network, and the mean KAM (~1.1°) remains comparable to CIP, indicating similarly high intragranular lattice curvature and GND density. Because the hysteresis coefficient K_h_ broadly tracks coercivity at fixed induction (i.e., K_h_ ∝ H_c_), the modest reduction in LAGB content—fewer deformation-induced barriers—translates into a small decrease in H_c_ and, accordingly, a slightly lower K_h_ for RIP relative to CIP. The higher HAGB fraction further subdivides domains and provides strong but more widely spaced pinning sites; combined with the preserved KAM, this yields a pinning landscape that is robust yet less tortuous than CIP’s dense subgrain network. Overall, the EBSD metrics (lower LAGB, similar KAM, higher HAGB) consistently explain RIP’s marginally reduced coercivity and its correspondingly moderate hysteresis loss.

CIP-P shows the lowest LAGB fraction and the highest HAGB fraction among the pure-Fe powders; the red LAGB network is visibly sparser, while blue HAGBs dominate. Its mean KAM (~1.0°) is slightly below that of CIP/RIP, indicating reduced intragranular lattice curvature at the subgrain scale. The phosphate coating serves as a critical buffer, mitigating the transfer of this mechanical stress between adjacent particles during compaction. By creating an interparticle barrier, the coating helps to prevent the formation of severe lattice strain and defects within the magnetic particles themselves [35]. Nevertheless, CIP-P exhibits a higher hysteresis loss. This arises from the type and location of pinning rather than the sheer amount of deformation substructure. The rigid chemical conversion phosphate shell and its thermal expansion mismatch with Fe concentrate residual magnetoelastic stresses at particle surfaces and interparticle bridges—features that EBSD KAM (an area-averaged measure) under-weights—further elevating H_c_. Surface P–O/oxide complexes at the metal–phosphate interface can also increase near-surface anisotropy and interface pinning [36]. Because the hysteresis coefficient K_h_ broadly tracks H_c_, these strong pinning and magnetoelastic effects outweigh the modest reduction in LAGB/KAM, yielding a higher K_h_ and thus higher hysteresis loss for CIP-P relative to CIP/RIP; with comparable particle size/insulation, the resulting increase in total core loss primarily reflects this hysteretic contribution.

FeSiCr displays coarser grains with a boundary network dominated by blue HAGBs—features largely inherited from atomization and solidification. Although its LAGB fraction (44.1%) is not the lowest, the grain interiors are comparatively strain-poor, as reflected by the lowest mean KAM (~0.7°) in the set, indicating that during compaction, FeSiCr densifies mainly via micro-fracture rather than extensive slip, thereby limiting micron-scale lattice curvature and dislocation storage captured by EBSD/KAM. In contrast, XRD shows the largest effective FWHM for FeSiCr, which is sensitive to nanoscale microstrain and coherent-domain refinement; this broadening can arise from solid-solution (Si/Cr)-induced elastic strain fields and statistically stored dislocations below the EBSD step size and thus does not contradict the low KAM [32,37]. Together, these results imply that FeSiCr stores a larger fraction of its press-induced strain in EBSD-sub-resolution fields (seen by XRD) while exhibiting limited long-wavelength curvature (seen by EBSD). Meanwhile, the elevated Si/Cr solute content introduces chemical pinning that impedes domain wall motion independent of stored strain. Because LAGBs are dislocation walls, a higher LAGB fraction generally raises the density of potential pinning sites, reinforcing the KAM–hysteresis linkage. The observation that CIP and RIP possess similar mean KAMs despite different LAGB shares explains why their $H_{c}$ and hysteresis losses are close: KAM sets the overall curvature (pinning intensity), whereas LAGB fraction modulates the spatial continuity of pinning.

Taken together, our data position RIP as a practical single-powder route that balances electromagnetic efficiency, durability, and sustainability. Its fine median size (≈4–6 µm) and continuous SiO_2_ shell provide strong electrical segmentation, expressed as a reduced eddy-current coefficient K_e_ across 200 kHz–1 MHz, while the hysteresis term K_h_ remains moderate despite plasticity-dominated compaction (mean KAM ≈ 1.1°); the net effect is a favorable total-loss profile compared with FeSiCr at high frequency. In salt-spray testing, RIP matches the corrosion robustness of silica-coated CIP and far outperforms phosphate-coated variants, adding a lifetime advantage that complements its loss benefits. From an environmental standpoint, the inert, phosphate-free SiO_2_ coating reduces reliance on acid-phosphate treatments and the associated wastewater, and RIP’s iron core is compatible with feedstocks derived from recycled iron oxides, lowering embodied resource intensity relative to alloyed FeSiCr. Although RIP exhibits somewhat lower μ_i_ than coarse FeSiCr due to increased domain wall pinning from its finer size/insulation, this trade-off is often acceptable where efficiency and service life dominate the design envelope. Overall, RIP offers a credible path to low-loss, corrosion-robust, and more sustainable molded inductors.

Table 4 shows the DC-bias performance at 15 A. The inductance-retention ranking is CIP (67.9%) > RIP (55.7%) > CIP-P (48.8%) > FeSiCr (33.2%), computed as L(15 A)/L_0_ = 1 − ΔL%. The effective anisotropy K_eff_ governing differential permeability under a DC offset comprises (i) magnetocrystalline/chemical anisotropy (e.g., Si/Cr in FeSiCr), (ii) magnetoelastic (stress) anisotropy from compaction and thermal mismatch, (iii) interfacial/surface anisotropy and pinning (e.g., phosphate-rich interfaces, particle contacts), and (iv) microstructural/defect anisotropy from deformation substructures (LAGB networks, GND density) together with interparticle demagnetizing fields. Lower K_eff_ broadens the field window for reversible domain wall bulging/glide, sustaining μ_d_ = dM/dH and thus L; high M_s_ keeps the magnetization curve farther from saturation at a given H_DC_, further preserving μ_d_.

Consistent with EBSD, CIP exhibits a dense LAGB/KAM substructure—this raises low-field hysteresis (K_h_) but, under DC bias, provides many soft, distributed barriers that enable reversible wall motion over a broad field range [38]; combined with the high M_s_ of pure Fe, CIP retains the highest L at 15 A. RIP shows a similar KAM but slightly fewer LAGBs and a finer particle size; the finer size increases interparticle demagnetizing fields and modestly broadens anisotropy dispersion [38], narrowing the bias-linear window relative to CIP. With M_s_ still high, RIP therefore shows intermediate retention. CIP-P has the lowest LAGB and highest HAGB fractions—fewer mild barriers but harder ones—and its phosphate interface concentrates magnetoelastic stress and near-surface anisotropy; domain walls pin/annihilate earlier, the response transitions to rotation sooner, and μ_d_ collapses earlier, yielding a larger drop than CIP/RIP. FeSiCr combines higher magnetocrystalline anisotropy and lower M_s_ with solute-induced pinning, producing the earliest roll-off and the largest inductance loss. Overall, the 15 A trends align with the EBSD/loss framework: microstructures and interfaces that keep K_eff_ low while preserving high M_s_ sustain inductance under DC excitation, whereas hard interfacial pinning or strong intrinsic anisotropy precipitate early roll-off.

This framework establishes a direct, quantitative linkage between microstructure and performance. Specifically, the high hysteresis loss coefficient (K_h_) in the ductile Fe powders is directly correlated with high mean lattice strain (mean KAM ≈ 1.0–1.1°), which indexes the dense dislocation substructures that pin domain walls. Conversely, the eddy-current coefficient (K_e_) is governed not by KAM but by particle size and insulation, with the fine (D50 ≈ 4–6 μm), silica-coated powders (CIP/RIP) showing the lowest K_e_. Furthermore, these substructures dictate DC-bias performance: the dense, soft LAGB network of CIP (LAGB ≈ 52%) facilitates reversible wall motion, yielding the highest 15 A inductance retention (67.9%). In contrast, the low KAM (≈0.7°) of FeSiCr is overshadowed by high chemical/magnetocrystalline anisotropy, resulting in the poorest retention (33.2%). Thus, mean KAM and LAGB fractions serve as direct quantitative proxies for the strain-induced component of K_h_ and the reversibility of domain wall motion under DC bias.

The microstructure–loss relationships established here are mechanism-based and therefore can be extrapolated beyond Fe and FeSiCr to Fe–based alloy type SMCs (e.g., FeSiAl, FeCrAl). In all cases, the eddy-current coefficient follows the classical scaling $K_{e}\propto \frac{D_{50}^{2}}{\rho_{eff}}$ modulated by insulation continuity; thus, finer particles and robust, electrically resistive shells yield lower $K_{e}\;$irrespective of chemistry. The hysteresis term,$\;K_{h},$ decomposes into (i) strain pinning, indexed by EBSD metrics (mean KAM, LAGB%) that quantify stored plastic strain/dislocation walls, and (ii) a solute/ordering-pinning baseline set by composition. Because Fe–based alloy powders are comparatively hard/brittle, pressing typically produces a lower KAM/LAGB (limited plasticity), so $K_{h}\;$is expected to be dominated by the solute/ordering contribution (Al/Si-induced magnetoelastic fields, potential antiphase boundary-related pinning), unless processing deliberately increases plastic flow (e.g., warm/forming routes). Practically, the functional mappings remain unchanged—$KAM/LAGB\;\to \;K_{h}$ and $(D_{50},\rho_{eff},and\;$shell continuity$)\;\to \;K_{e}$—while the coefficients should be recalibrated to the alloy family’s $M_{s}$, $K_{1}$, $\lambda_{s}$, and resistivity.

## 4. Conclusions

We establish a quantitative, microstructure-informed basis for selecting soft magnetic powders for 100 kHz–1 MHz molded inductors. In the loss model P(f) = K_h_f + K_e_f^2^, the hysteresis coefficient K_h_ increases with plastic deformation and stored lattice strain (indexed by mean KAM and LAGB fraction): ductile Fe powders (CIP, RIP) develop dense subgrain networks that elevate K_h_, whereas FeSiCr, despite its rearrangement-dominated compaction and low KAM, still exhibits high K_h_ due to Si/Cr-solute pinning of domain walls. The eddy-current term K_e_ is governed by particle size and insulation continuity, with fine, silica-coated powders (CIP, RIP) suppressing K_e_ relative to coarse, uncoated FeSiCr. Coating chemistry also dictates durability (SiO_2_ ≫ phosphate), while Cr in FeSiCr aids passivation.

These relationships translate directly into design principles for molded cores: limit excessive plastic flow during compaction to restrain subgrain formation and K_h_; select fine powders with continuous inorganic shells to minimize K_e_; and prioritize robust SiO_2_-based insulation (with Cr-assisted alloy passivation where relevant) to ensure corrosion resistance and lifetime stability. Within this framework, reduced-iron powder (RIP) provides a balanced and sustainable choice—its fine size and continuous SiO_2_ shell lower K_e_, K_h_ remains moderate, corrosion robustness is comparable to CIP, and the phosphate-free coating together with compatibility with recycled Fe feedstocks reduces environmental burden. At 15 A, inductance retention ranks CIP (67.9%) > RIP (55.7%) > CIP-P (48.8%) > FeSiCr (33.2%). CIP leads because high M_s_ and a dense but soft LAGB/KAM substructure keep K_eff_ low enough to sustain reversible wall motion under bias, whereas FeSiCr rolls off earliest due to higher magnetocrystalline anisotropy and lower M_s_. RIP is intermediate (slightly fewer LAGBs and stronger interparticle demagnetizing fields), while CIP-P lags CIP/RIP as phosphate-related interfacial stress/anisotropy and a higher HAGB fraction promote earlier wall immobilization. Collectively, these criteria position RIP as a credible single-powder route to low-loss, corrosion-robust molded inductors across the 100 kHz–1 MHz band.

## Figures and Tables

**Table 1:** XRF compositions of raw powders (wt%).

Powder	Fe (wt%)	Si (wt%)	Cr (wt%)	P (wt%)	Remarks
FeSiCr (matrix)	88.5	5.1	6.1	n.d.	Fe–Si–Cr solid solution
RIP (SiO_2_-coated)	>98	trace	n.d.	n.d.	Si from silica shell
CIP (SiO_2_-coated)	>98	trace	n.d.	n.d.	Si from silica shell
CIP-P (phosphate-coated)	>98	n.d.	n.d.	trace	P from phosphate shell

n.d. = not detected above the instrument’s reporting limit.

**Table 2:** Particle-size distribution metrics (laser diffraction).

Powder	D_10_ (µm)	D_50_ (µm)	D_90_ (µm)	Span = (D_90_ − D_10_)/D_50_
FeSiCr	12.4	17.8	30.6	1.02
RIP	2.4	4.6	9.1	1.46
CIP	2.7	5.5	9.8	1.29
CIP-P	2.5	4.9	9	1.33

**Table 3:** Relative compaction density (%) of T-cores pressed at 200 Mpa.

Powder (T-Core)	Relative Compaction Density (%)
CIP	74.4
CIP-P	73.9
RIP	72.3
FeSiCr alloy	71.7

**Table 4:** Inductance (L) retention after applying DC bias current of 15A.

Sample	Retention of L at 15 A (%)
CIP-P	48.8
CIP	67.9
RIP	55.7
FeSiCr	33.2

## Data Availability

The original contributions presented in this study are included in the article/Supplementary Material. Further inquiries can be directed to the corresponding author.

## Supplementary Materials

The following supporting information can be downloaded at: https://www.mdpi.com/article/10.3390/ma18215016/s1, Figure S1: Volume-based particle-size distribution (PSD) curves of the raw materials. Figure S2: XRD patterns of the raw materials, as received powders (left column) and compacts after pressing at 200 MPa (right column).
