# Supplementary material for: Microstructure (EBSD-KAM)-Informed Selection of Single-Powder Soft Magnetics for Molded Inductors
Source: Materials (Basel). 2025 Nov 4;18(21):5016. doi: 10.3390/ma18215016 (PMC12608291; doi:10.3390/ma18215016)
Supplement: Supplementary file 1 [file materials-18-05016-s001.zip › materials-3941101-supplementary.pdf]

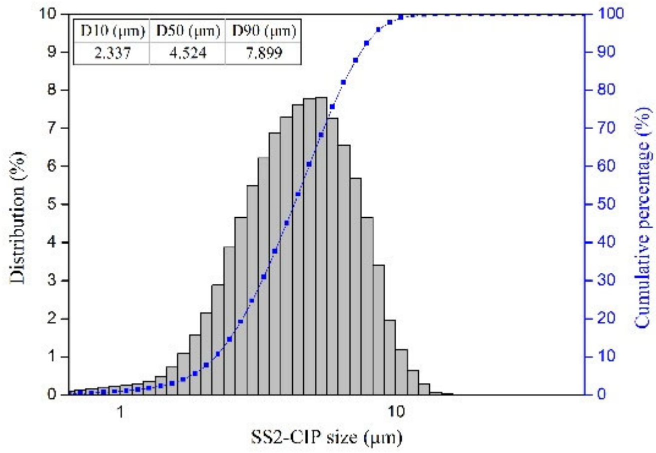


CIP

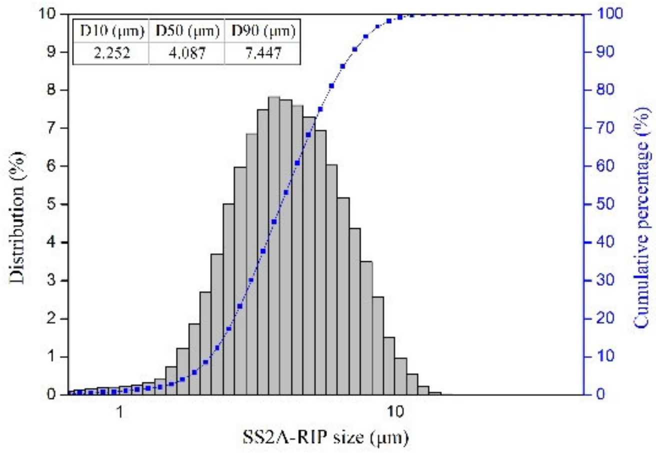


RIP

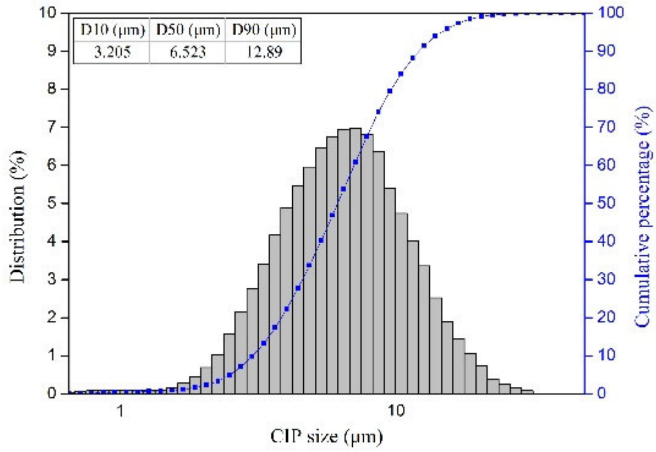


CIP-P

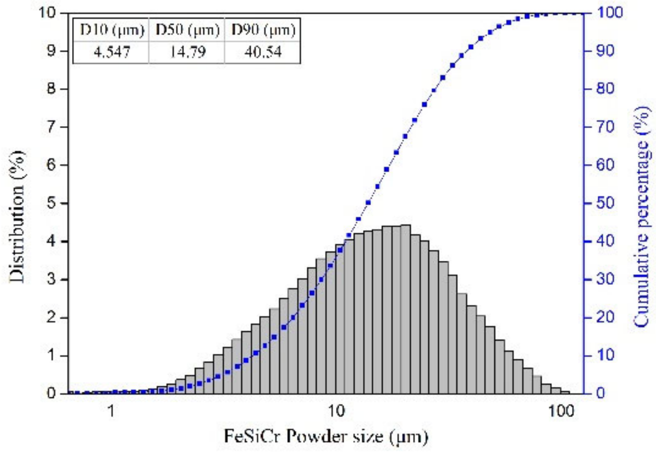


FeSiCr

Figure S1 Volume-based particle-size distribution (PSD) curves of the raw materials.

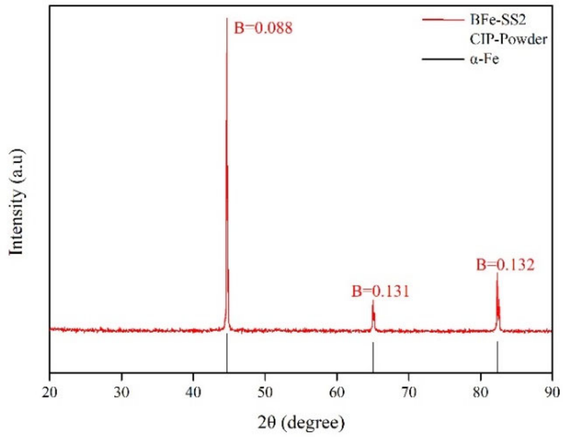


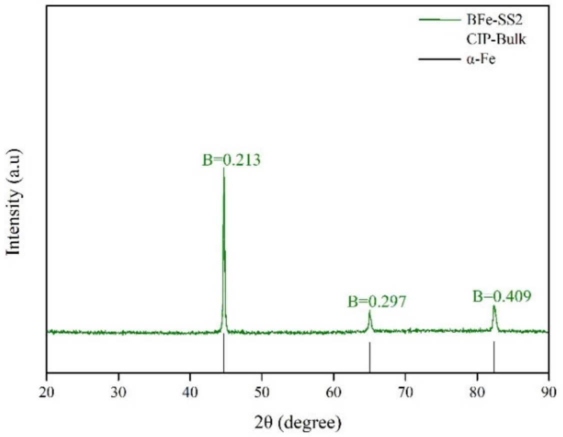

CIP

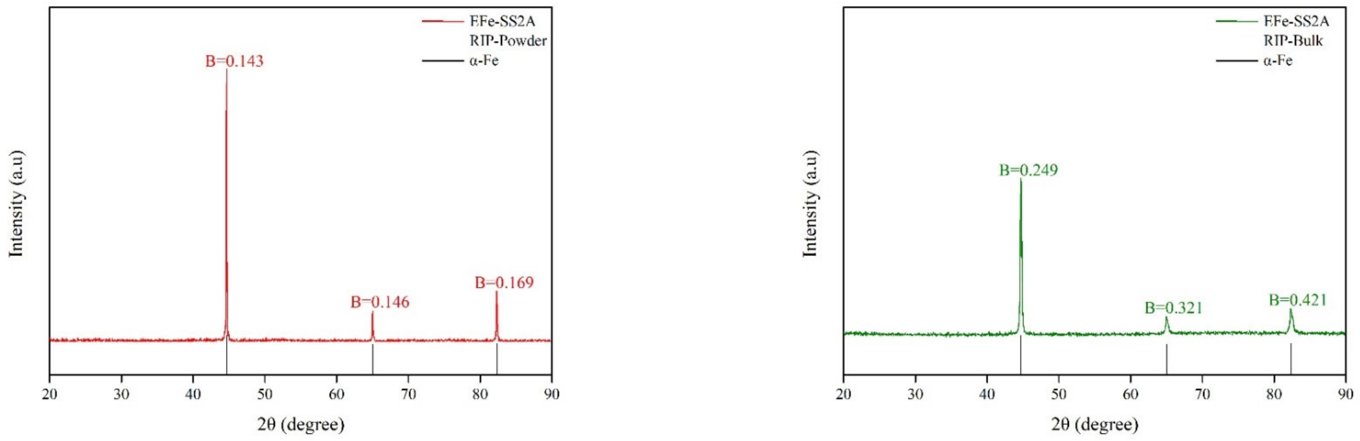


RIP

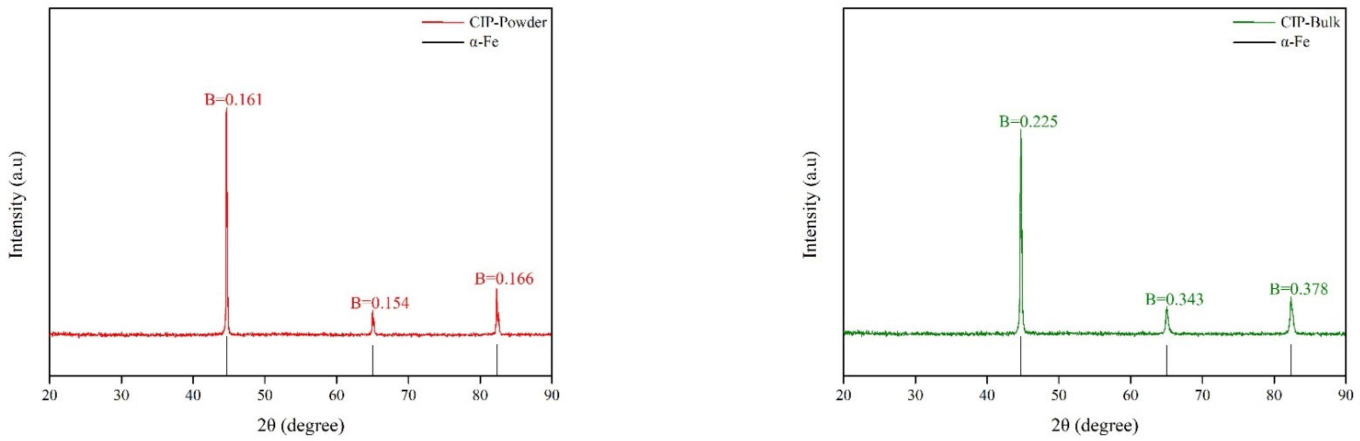


CIP-P

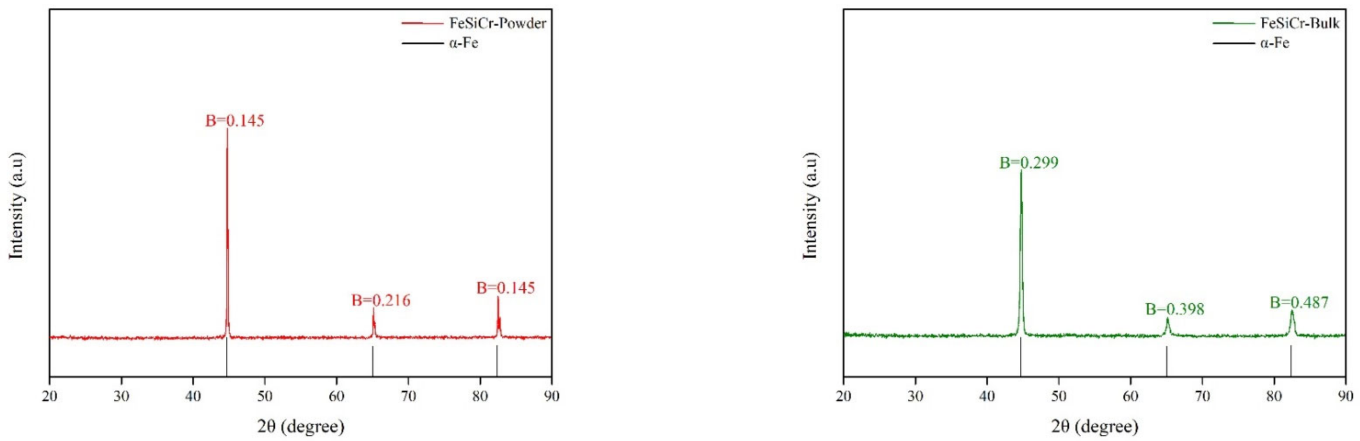


FeSiCr

Figure S2 XRD patterns of the raw materials, as received powders (left

column) and compacts after pressing at 200 MPa (right column).
